# Azarshahr travertine compression strength prediction based on point-load index (I_s_) data using multilayer perceptron

**DOI:** 10.1038/s41598-023-46219-4

**Published:** 2023-11-27

**Authors:** Yimin Mao, Zhu Licai, Li Feng, Yaser A. Nanehkaran, Maosheng Zhang

**Affiliations:** 1 School of Information and Engineering, Shaoguan University, Shaoguan, 512005 Guangdong China; 2 School of Information Engineering, Yancheng Teachers University, Yancheng, 224002 Jiangsu People’s Republic of China; 3 School of Human Settlement Environment and Civil Engineering, Xi’an Jiaotong University, Xi’an, 712000 Shaanxi China

**Keywords:** Natural hazards, Planetary science, Solid Earth sciences, Engineering, Materials science

## Abstract

Azarshahr County in the northwest of Iran is predominantly covered by Azarshahr travertine, a prevailing sedimentary rock. This geological composition has led to extensive open-pit mining activities, particularly in the western and southwestern parts of the county. The rock's drillability and resistance to excavation play a pivotal role in determining its overall durability and hardness, crucial factors that influence the mining process. These attributes are intimately tied to the compressive strength of the rock. Accurate assessment of rock strength is vital for devising reliable excavation methodologies at mining sites. However, conventional approaches for analyzing rock strength have limitations that undermine the precision of strength estimations. In response, this study endeavors to leverage artificial intelligence techniques, specifically the Multilayer Perceptron (MLP), to enhance the prediction of travertine's compressive strength. To formulate a robust model, a comprehensive database containing data from 150 point-load index (I_s_) tests on Azarshahr travertine was compiled. This dataset serves as the foundation for the development of the MLP-based predictive model, which proves instrumental in projecting rock compressive strength. The model's accuracy and efficacy were rigorously assessed using the Receiver Operating Characteristic (ROC) curve, employing both training and testing datasets. The modeling outcomes reveal impressive results. The estimated R-squared coefficient attained an impressive value of 0.975 for axial strength and 0.975 for diametral strength. The overall accuracy, as indicated by the Area Under the Curve (AUC) metric, stands at an impressive 0.968. These exceptional performance metrics underscore the efficacy of the MLP model in accurately predicting compressive strength based on the point-load index of samples. The implications of this study are substantial. The predictive model, empowered by the MLP approach, has profound implications for excavation planning and drillability assessment within the studied region's travertine deposits. By facilitating accurate forecasts of rock strength, this model equips mining endeavors with valuable insights for effective planning and execution.

## Introduction

Travertine, classified as a type of sedimentary rock, emerges through the precipitation of calcium carbonate from water, commonly occurring within limestone caves or hot springs. Its composition primarily comprises minerals such as calcite, aragonite, and diverse forms of calcium carbonate, occasionally accompanied by trace amounts of organic matter^1^. Notably porous in texture, travertine generally exhibits a light hue, spanning from white to beige. Its versatile attributes render travertine a sought-after building material, serving numerous applications both indoors and outdoors. It finds use in flooring, wall cladding, countertops, as well as ornamental elements like columns, sculptures, and fountains. Its historical significance traces back to ancient Roman times, where it garnered favour due to its resilience and resistance against weathering^2^. Beyond durability, travertine's appeal lies in its distinct aesthetic allure. While classified as a form of limestone, travertine distinguishes itself by forming through mineral precipitation from groundwater rather than the accumulation of organic matter. Its presence extends across various nations, including Italy, Turkey, Mexico, and the United States, often located in proximity to mineral-rich water sources and hot springs^1^. The rock's unique colour variations, veining patterns, and textures stem from the diverse geological conditions under which it is created^2^. Travertine's durability stands as a key advantage in the realm of construction. It can endure elevated temperatures, heavy foot traffic, and the effects of weathering. Yet, its porous nature necessitates regular sealing to safeguard against moisture-induced damage and staining. However, travertine's utility extends beyond construction purposes. It contributes to the production of lime and cement, serves as an agricultural soil conditioner, and adds an ornamental touch to landscaping endeavours^3^.

From a geotechnical perspective, travertine exhibits remarkable resilience against a broad spectrum of weather conditions and environmental elements. Its enduring nature makes it a prime choice for an extensive array of indoor and outdoor applications, encompassing flooring, countertops, wall cladding, and exterior paving^3^. Despite its exceptional durability, however, travertine retains its porous nature, rendering it susceptible to the absorption of liquids and various substances. Failure to adequately seal and maintain the rock can result in stains and damage. Regular cleansing and the application of a sealant are imperative to safeguard travertine from moisture and other environmental factors, preserving its longevity^4^. In essence, when properly cared for, travertine stands as a markedly durable and enduring natural stone, capable of elevating the aesthetics and value of any structure or residence^3^. Consequently, travertine finds utility in construction materials, ground modification, architectural stones, and more^5^. Nevertheless, regardless of these advantages, drillability and rock resistance during excavation are of paramount significance in open-pit mining endeavours. This determination shapes effective excavation strategies for extracting travertine blocks. Drillability, specifically, pertains to the ease or difficulty of drilling through a particular rock type utilizing drilling tools. The drillability of rock hinges on numerous factors, encompassing rock type, hardness, porosity, fractures, and the nature of the drilling apparatus employed^6^. In various industries such as mining, construction, and geotechnical engineering, rock drillability significantly affects both drilling efficiency and costs. For instance, a rock that permits easy drilling may expedite mineral extraction or tunnel construction, while a dense and hard rock could necessitate specialized tools and techniques^7,8^. Evaluating rock drillability involves the drillability index, a metric gauging drilling rate and energy required for penetrating a rock sample under controlled conditions^6^. Additional factors influencing this index include fracture orientation, spacing, groundwater presence, temperature, and pressure. Accurate estimation of rock compressive strength proves invaluable in devising dependable excavation methodologies at mining sites.

**Figure 1:**
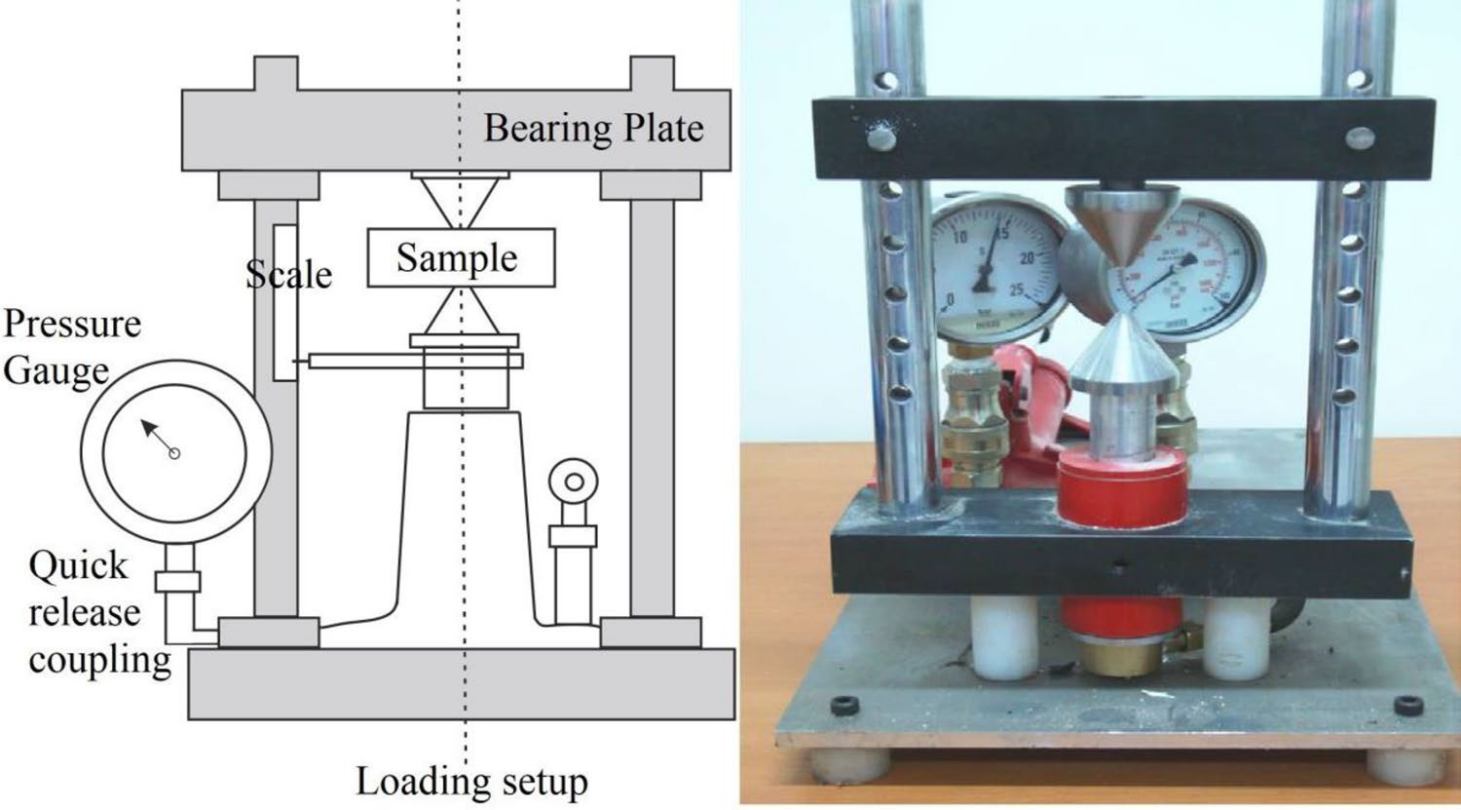
A scheme of a point-load test^17^.

The estimation of rock compressive strength typically involves various geotechnical in-situ or laboratory tests, including the uniaxial compressive strength (UCS) test^9^, Schmidt hammer test^10^, point-load test^11^, beam bending test^12^, ring shear test^13^, and triaxial compression test^14^. Among these, both UCS and point-load tests play a pivotal role in ore crusher design and the evaluation of rock strength for mining purposes. Notably, the advantages inherent in the point-load test make it a more appealing method compared to the UCS approach. Its efficacy in estimating rock compressive strength is particularly prominent when applied in both field and laboratory settings^15^. The point-load test, a widely used method for measuring rock strength, entails the application of a specific load to a small cylindrical rock sample through a device known as a point-load tester^16^. The testing procedure involves positioning the rock sample between two pointed platens. A load is directed onto one of these platens, in accordance with the guidelines outlined by the American Society for Testing Materials (ASTM)^11^ and the International Society of Rock Mechanics (ISRM). As the load gradually increases, the force needed to fracture the sample is measured until the rock sample ultimately breaks. The point-load strength index (I_s_), serving as an indicator of the rock's strength, is then determined by considering the force necessary for fracturing the sample. For visual clarification, refer to Fig. 1, which presents an illustration of the point-load test^17^.

The point load test is a foundational geotechnical evaluation technique employed to assess the mechanical strength of rocks. This method involves subjecting a rock sample to a concentrated load at a specific point on its surface, measuring the force required to induce fracture, and calculating the resulting point load index (I_s_). This index serves as an indicator of the rock's strength and plays a crucial role in various engineering and mining applications. Point load tests are particularly advantageous due to their simplicity, speed, and versatility^17^. They offer rapid results and can be conducted in both field and laboratory settings, making them suitable for a wide array of scenarios. Furthermore, point load tests provide localized insights into a rock's strength under specific stress conditions, serving as a preliminary assessment that aids in determining the need for further, more complex testing. However, point load tests have inherent limitations. They offer a focused assessment and may not fully capture the intricate variability or anisotropic nature of certain rock formations. The results are sensitive to specimen dimensions and geometries, necessitating careful consideration^16^. In spite of these constraints, point load tests remain a cost-effective and efficient tool for promptly obtaining indicative information about rock mechanics, facilitating informed decisions across engineering and mining sectors.

The point-load test is generally regarded as a valuable tool in geotechnical investigations due to its capacity for on-site application, as well as its relative simplicity, speed, and cost-effectiveness^16^. Drawing upon a comprehensive review of relevant literature, as summarized in Table 1, the background of the point-load test reveals significant insights. Broch and Franklin^18^ pioneered the development of the inaugural point-load strength formula through damage model analysis of cylindrical specimens. Bieniawski^19^ affirmed a direct correlation between rock compressive strength and the point load index (I_s_), suggesting empirical methods for its estimation. Tsiambaos and Sabatakakis^20^ delved into I_s_ variations within sedimentary rock, culminating in specific relationships tailored to this category of rock materials. Addressing anisotropic rocks, Basu and Kamran^21^ established an empirical link to assess rock strength through estimated I_s_ values. Analyzing the contents of Table 1 underscores the extensive utilization of I_s_ and point load results for the estimation of rock compressive strength. Consequently, the formulation of accurate relationships can significantly contribute to an enhanced comprehension of the strength attributes of rock materials.

**Table 1:** A summary of several works on UCS and I_s_ empirical relationship.

Relation	Rock type	Method	Scholar(s)/year	Reference
UCS = 15.3 I_s(50)_ + 16.3	General	Empirical	D’andrea et al./1965	^22^
UCS = 20.7 I_s(50)_ + 29.6	General	Empirical	Deer and Miller /1966	^23^
UCS = 23.7 I_s(50)_	General	Empirical	Broch and Franklin/1972	^24^
UCS = 23.9 I_s(50)_	Sedimentary	Empirical	Bieniawski/1975	^25^
UCS = 29 I_s(50)_	Sedimentary	Empirical	Hassani et al./1980	^26^
UCS = 20 I_s(50)_	Sedimentary	Empirical	Read et al./1980	^27^
UCS = 18.7 I_s(50)_—13.2	General	Empirical	Singh /1981	^28^
UCS = 14 I_s(50)_	General	Empirical	Forster/1983	^29^
UCS = 16.5 I_s(50)_ + 51.0	General	Empirical	Gunsallus and Kulhawy/1984	^30^
UCS = 20–25 I_s(50)_	General	Empirical	ISRM/1985	^31^
UCS = 12.6–18 I_s(50)_	Sedimentary	Empirical	Das/1985	^32^
UCS = 24.8–26.5 I_s(50)_	Sedimentary	Empirical	Hawkins and Olver /1986	^33^
UCS = 30 I_s(50)_	Sedimentary	Empirical	O’Rourke/1988	^34^
UCS = 12.6 I_s(50)_	Igneous	Empirical	Vallejo et al./1989	^35^
UCS = 14–82 I_s(50)_	General	Empirical	Cargill and Shakoor/1990	^36^
UCS = 16 I_s(50)_	Igneous	Empirical	Ghosh and Srivastava/1991	^37^
UCS = 9.30 I_s(50)_ + 20.04	General	Empirical	Grasso et al./1992	^38^
UCS = 19 I_s(50)_ + 12.7	Sedimentary	Empirical	Ulusay et al./1994	^39^
UCS = 8.41 I_s(50)_ + 9.51	General	Empirical	Kahraman/2001	^40^
UCS = 3.86 [I_s(50)_]^2^ + 5.65 I_s(50)_	Volcanic	Empirical	Quane and Russell/2003	^41^
UCS = 7.3 [I_s(50)_]^1.71^	Sedimentary	Empirical	Tsiambaos et al./2004	^20^
UCS = 9.08 I_s(50)_ + 39.32	General	Empirical	Fener et al./2005	^42^
UCS = 8 I_s(50)_	Sedimentary	Empirical	Sabatakakis et al./2008	^43^
UCS = 16.45 e^0.39Is(50)^	Metamorphic	Empirical	Diamantis et al./2009	^44^
UCS = 11.103 I_s(50)_ + 37.66	Igneous	Empirical	Basu et al./2010	^21^
UCS = 5.57 I_s(50)_ + 21.92	Gypsum	Empirical	Heidari et al./2012	^45^
UCS = 7.73 [I_s(50)_]^1.25^	Volcanic	Empirical	Kahraman/2014	^46^
UCS = -0.66 [I_s(50)_]^2^ + 21.15 I_s(50)_	Igneous	Empirical	Zhang et al./2015	^47^
UCS = 18.897 I_s(50)_	Volcanic	Empirical	Wong et al./2017	^48^
UCS = 22.72–26.24 [I_s(50)_]^m^	Sedimentary	Empirical	Chen and Wei/2018	^49^
UCS = 21.28 I_s(50)_	Metamorphic	Empirical	Li et al./2019	^50^
UCS = 24.3–2.48 I_s(50)_ + 6.4 ~ 8.05	Sedimentary	Empirical	The authors	This study

The point load test on rocks presents several advantages. Firstly, it offers a swift and uncomplicated method, suitable for both field and laboratory settings, to assess rock strength. This enables preliminary evaluations of rocks' suitability for construction or mining activities. The non-destructive nature of the test allows for repeated measurements on the same sample, aiding in data collection efficiency. Additionally, the direct correlation between applied load and strength index simplifies interpretation, and the test serves well for comparative analyses between different rock types. The point load test also has limitations to consider. While the test's speediness is an asset, it provides localized strength values that might not accurately reflect the overall rock mass strength. Variability can arise due to specimen characteristics, such as shape and texture. Anisotropic properties of rocks are not accounted for, and the test's focus on compressive strength neglects other vital mechanical attributes. The presence of fractures or weak planes can influence results, potentially leading to inaccuracies. Thus, while the point load test offers valuable insights, its interpretation should acknowledge these limitations, and it should complement more comprehensive testing methods for precise engineering applications^20,21,42–50^.

In recent times, the evolution of technology has prompted a notable interest in the adoption of more intricate approaches, including artificial intelligence (AI) and computational methods. The integration of AI-based techniques in predicting rock strength through the analysis of diverse geological data has yielded expedited, reliable, and highly accurate outcomes^51^. Employing machine learning algorithms, AI techniques offer the capability to process vast geological datasets and forecast rock strength. These algorithms have the capacity to assimilate historical data from rock samples, encompassing the rock's physical characteristics and mineral composition. Subsequently, they can predict the strength of new rock samples by recognizing patterns extracted from the amassed data^52^. The recent advancements in this domain underscore the potential of AI as a potent tool for prognosticating rock strength, carrying significant implications across geotechnical engineering, mining, and related fields^51^. The predictive potential of AI bestows several advantages over traditional methodologies, including heightened precision and accuracy, expedited results, cost-effectiveness, non-destructiveness, and adaptability to diverse types of rock materials^52^.

In essence, the fundamental aim of AI and machine learning models is to offer heightened accuracy in results while simultaneously striving to reduce process costs. Traditional methodologies for predicting rock strength often entail physical testing, a procedure that can be both time-consuming and financially burdensome. In contrast, intelligent models hold the potential to swiftly forecast rock strength while also curtailing examination expenditures. Machine learning models exhibit a cost-effectiveness that frequently surpasses that of conventional testing methods, especially when considering the expenses associated with equipment, time, and personnel required for physical testing. This efficiency opens the door to rock strength predictions on a broader scale, enabling extensive surveys and the execution of a significant volume of tests. Furthermore, traditional rock strength testing typically demands destructive examinations involving bore-holing, coring, sampling, and rigorous testing procedures. In contrast, predictive models can yield results with minimal invasiveness, contributing to reduced expectations. Notably, AI-based models operate independently of specific databases, rendering them versatile and unaffected by shifts in material types. This adaptability positions them as viable tools for a range of geo-materials^51^.

The application of AI techniques to predict point load strength in rocks offers a multitude of advantages. Firstly, AI models leverage sophisticated algorithms to provide highly accurate predictions by discerning intricate relationships within complex rock datasets. This capability enables a more comprehensive understanding of rock behavior, particularly when dealing with nonlinear patterns and interdependencies among rock properties. Moreover, AI models adapt to new data, ensuring continuous enhancement in prediction accuracy and reliability over time. This adaptability translates into reduced testing costs and quicker decision-making by minimizing the need for extensive laboratory testing of rock samples. The time efficiency of AI-driven predictions is a valuable asset, particularly for real-time scenarios and time-sensitive projects. Additionally, AI's versatility extends to various rock types and geological conditions, making it an adaptable tool for geotechnical engineering and exploration activities. Furthermore, AI's data-driven insights provide a deeper understanding of factors influencing point load strength, facilitating better resource allocation and optimized operations in drilling, mining, and construction endeavors. Lastly, the objective nature of AI algorithms mitigates human bias, leading to impartial and reliable prediction outcomes. Incorporating AI into point load rock prediction thus enables geotechnical professionals to make informed decisions, streamline operations, and gain valuable insights into rock mechanics, contributing to safer and more efficient engineering projects.

The present study embarks on a novel approach, harnessing the power of the Multilayer Perceptron (MLP) network, a distinguished artificial neural network (ANN) technique renowned for its predictive, classification, and regression capabilities. This methodology not only introduces a fresh perspective but also holds substantial advantages in terms of accuracy and efficiency. The innovative aspect lies in its application to forecast the compressive strength of sedimentary rocks, with a specific focus on travertine. This unique application of the MLP model to predict rock strength based on the point-load index (I_s_) data from the Azarshahr region in the northwest of Iran demonstrates the study's pioneering nature. By employing this advanced technique, the study aims to achieve more accurate and rapid predictions compared to traditional methods. The utilization of MLP has the potential to capture intricate patterns within the data, thus enhancing the precision of the compressive strength forecasts. This approach stands as a testament to the study's cutting-edge methodology and its potential to yield valuable insights for geotechnical and mining applications.

## Methods and materials

### Point-load test basics

The act of applying an axial load to a precise point or region on the rock's surface is known as point-loading, forming the fundamental principle of the point-load test. This loading procedure endures until the rock experiences failure or the sample is compromised. By closely monitoring this progression, the compressive strength of the rock can be determined through empirical correlations. Among the concentrated loading techniques, diametral and axial point load strength is the most prevalent. These approaches are developed based on the analysis of rock conditions^31^, a recommendation stipulated by the International Society for Rock Mechanics (ISRM). Notably, the ISRM provides the relationship for the strength index denoted as I_s(50)_ for a 50 mm diameter, facilitating the calculation process:

1 $$ I_{s} = \frac{P}{{D_{e}^{2} }} $$

**Figure 2:**
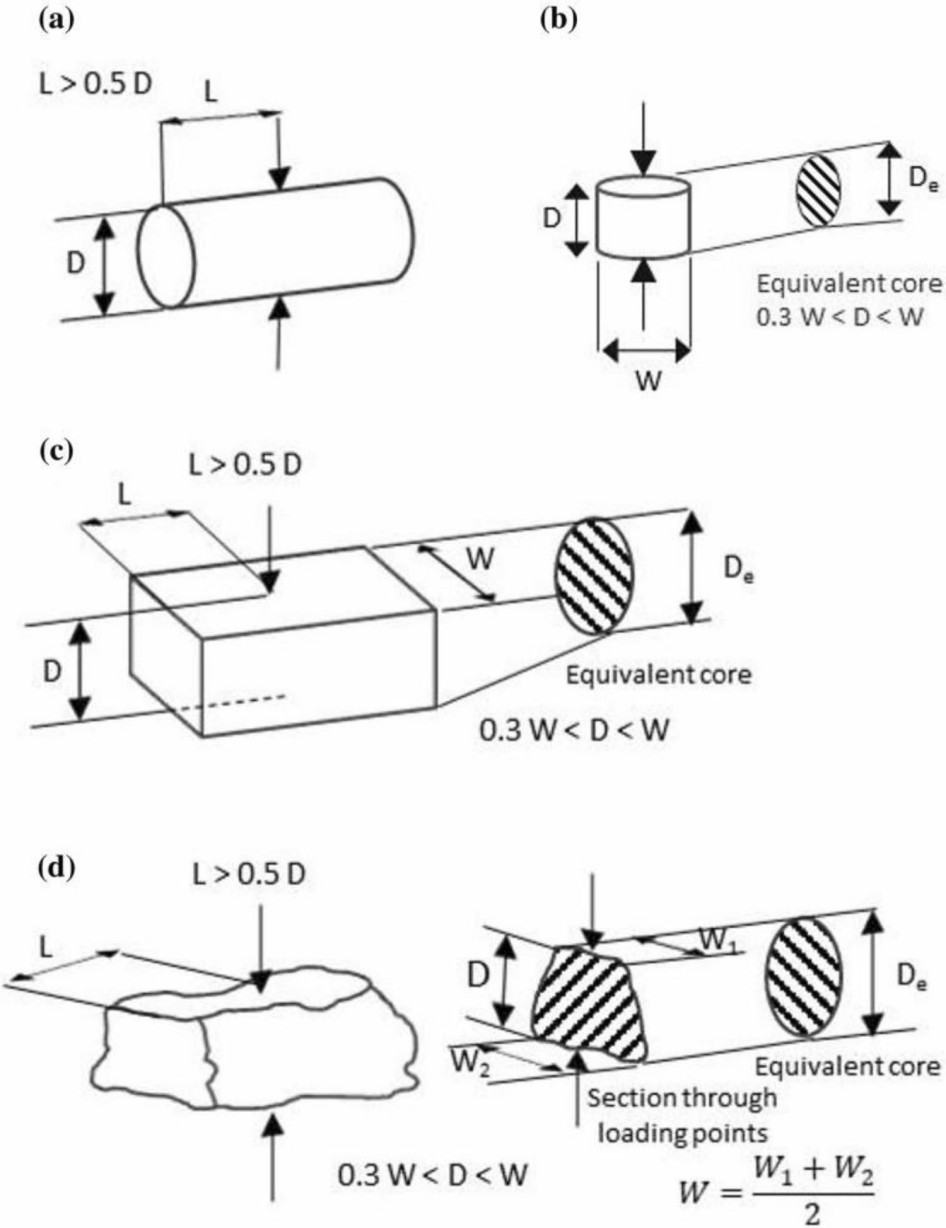
The geometry and dimension of various samples of rock: (**a**) diametral, (**b**) axial, (**c**) blocky (adapted from ISRM^31^ and^53^).

In this context, Is symbolizes the point-load index, while De stands for the equivalent sample diameter (mm), as stipulated by ISRM guidelines, and P signifies the applied loading. However, it's worth noting that access to the precise diameter value can sometimes be constrained. In recognition of this, the ISRM has introduced a corrective factor, rendering I_s(50)_ = αI_s_, as elucidated in Eq. (2).

2 $$ \alpha = \left( {\frac{{D_{e} }}{50}} \right)^{F} $$

where F is correction index, which is typically desired to be between 0.40 to 0.45, or based on the value of comparable rocks to determine experience. Presented study used lab equivalent core diameter (D) were calculated as

3 $$ D_{e} = \sqrt {\frac{4WD}{\pi }} $$

where D is loading point spacing, W is the average width across the two loading points for the smallest section ([W_1_ + W_2_]/2). Regarding the estimation of UCS, the ISRM^31^ was used. The results of the predictive models are setup based on ISRM regulations as well. That mean all experimental tests for estimation of input parameters such as UCS and I_s_ are controlled and verified based on ISRM instructions. These parameters have been shown in Fig. 2. The diameter of a rock sample is a critical factor in the determination of the I_s_ during point load tests. The impact of rock sample diameter on I_s_ is multifaceted and must be carefully considered for accurate and meaningful test results. When the diameter is increased, the load applied during the test is distributed over a larger surface area of the rock specimen. This distribution can lead to reduced stress concentration at the loading point, potentially resulting in a lower force required to cause fracture. Consequently, the calculated I_s_ may be lower for larger diameter samples even if the rock's inherent strength remains constant^26^. Moreover, the scale effect comes into play when evaluating different sample diameters. Smaller diameter samples might not capture the full complexity of the rock's structural characteristics, potentially yielding higher apparent strength due to the presence of fewer defects or fractures. On the other hand, larger diameter samples have a greater likelihood of representing the rock's bulk properties and inherent heterogeneity more accurately. However, this also introduces the challenge of effectively dealing with the anisotropic and heterogeneous nature of larger samples. The sample diameter's influence on I_s_ is also intertwined with the representative nature of the testing and its practical implications. Smaller samples might be easier to handle and test, but their results might not reflect the behaviour of larger rock masses^33–37^. Conversely, larger samples can offer insights into the broader mechanical behaviour of the rock, but they may require specialized testing equipment and adjustments to ensure accurate results. Ultimately, the choice of sample diameter should be aligned with the specific goals of the testing, the scale of the geological feature under study, and the balance between capturing realistic behaviour and practical implementation^53^.

### Rock sampling and data preparation

**Figure 3:**
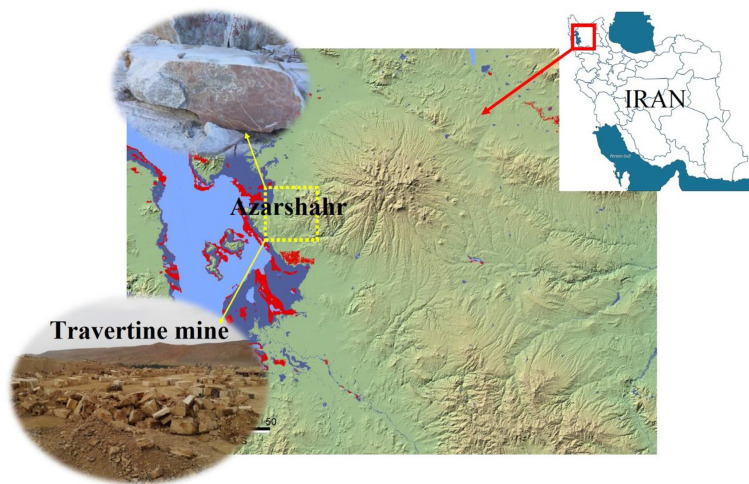
Location of the studied area in Iran.

To establish a comprehensive relationship between the I_s_ index and rock strength, an extensive set of 150 point-load tests were meticulously executed on samples extracted from Azarshahr travertine mines. These specimens, sourced from surface samples, were subjected to examination using a point load strength tester device. A geographical overview of the study area can be found in Fig. 3, pinpointing the location context. The original rock samples underwent a transition to the geotechnical laboratory setting. The estimation of I_s_ was undertaken following the guidelines set forth by ASTM^11^ and ISRM^31^. All samples underwent a process of coring and meticulous preparation in alignment with the necessary prerequisites for test conduction, as stipulated by the standards^11^. The samples designated for testing post-coring were maintained in a dry environment within the laboratory, ensuring dry conditions. Subsequently, the tests were executed under room temperature conditions. The dryness of the samples was ensured through rigorous control measures, attesting to the precision of the experimental conditions. The focal point of the analysis revolves around Azarshahr travertine, a distinct type of sedimentary rock categorized as calcium carbonate, specifically limestone. Upon transfer to the laboratory, the procured specimens were carefully drilled and standardized, resulting in cylindrical samples with a diameter of 10 × 5 cm, adhering to the regulations laid out by ISRM. The test procedure meticulously followed the instructions delineated by ISRM guidelines. Notably, each initial sample was subjected to testing on three separate occasions, a process undertaken with utmost precision and care. The resultant outcomes were tabulated and subjected to thorough analysis, with the average values across these tests being considered as representative results.

In the context of this study aimed at predicting the point-load strength index (I_s_) for rock samples, the absence of activation functions within the neural network architecture would have undermined the core objective. The goal was to establish a predictive model capable of capturing complex relationships between I_s_ and rock compressive strength (UCS) using an MLP. Activation functions played a pivotal role in enabling the model to learn and represent these intricate dependencies within the dataset, aligning perfectly with the research aim of constructing an accurate predictive model. The number of input features in an MLP model for point-load testing is a pivotal factor. The choice of relevant features significantly influences the model's performance and predictive accuracy. Selecting an inadequate number of input features may lead to an incomplete representation of the data, resulting in suboptimal predictions. Conversely, an excessive number of features could introduce noise and hinder the model's ability to generalize. Careful feature selection and dimensionality reduction techniques are essential to strike the right balance and ensure the MLP effectively captures the relevant information needed for accurate point-load predictions. The I_s_ value and UCS are the main features that we considered in this study.

Regarding the number of input features, the study's aim was to create an efficient predictive model for point-load tests, emphasizing the importance of selecting relevant input features for optimal predictions. The model's performance was closely tied to the choice of input features and their ability to represent critical information. The results showcased the effectiveness of the carefully chosen input features in achieving commendable accuracy and reliability in predicting I_s_ and UCS values. Therefore, within the context of this research, the presence of appropriate activation functions and the selection of relevant input features were pivotal in attaining the desired predictive outcomes.

### Machine learning model basics

An artificial neural network (ANN) variant called the multilayer perceptron (MLP) consists of several layers of nodes, each representing a straightforward mathematical function. These nodes are interconnected with weighted connections, where the outputs from one layer become inputs for the next. An MLP generally comprises an input layer, one or more hidden layers, and an output layer^51^. The input layer accepts input data, which then undergo processing through the hidden layers' nodes before ultimately being emitted through the output layer. Within the hidden layers, every node applies a mathematical function to its input before transmitting the output to the subsequent layer. A distinguishing hallmark of the MLP is that the connections' weights are refined through a process known as training. In this training phase, the network is provided with sets of input–output pairs, and the weights are adjusted to minimize the discrepancy between predicted and actual outputs. This adaptation typically involves employing optimization techniques like gradient descent^52^. The MLP stands as a robust tool for capturing intricate relationships between input and output, capable of being trained to perform tasks beyond the scope of traditional methods^51^.

**Figure 4:**
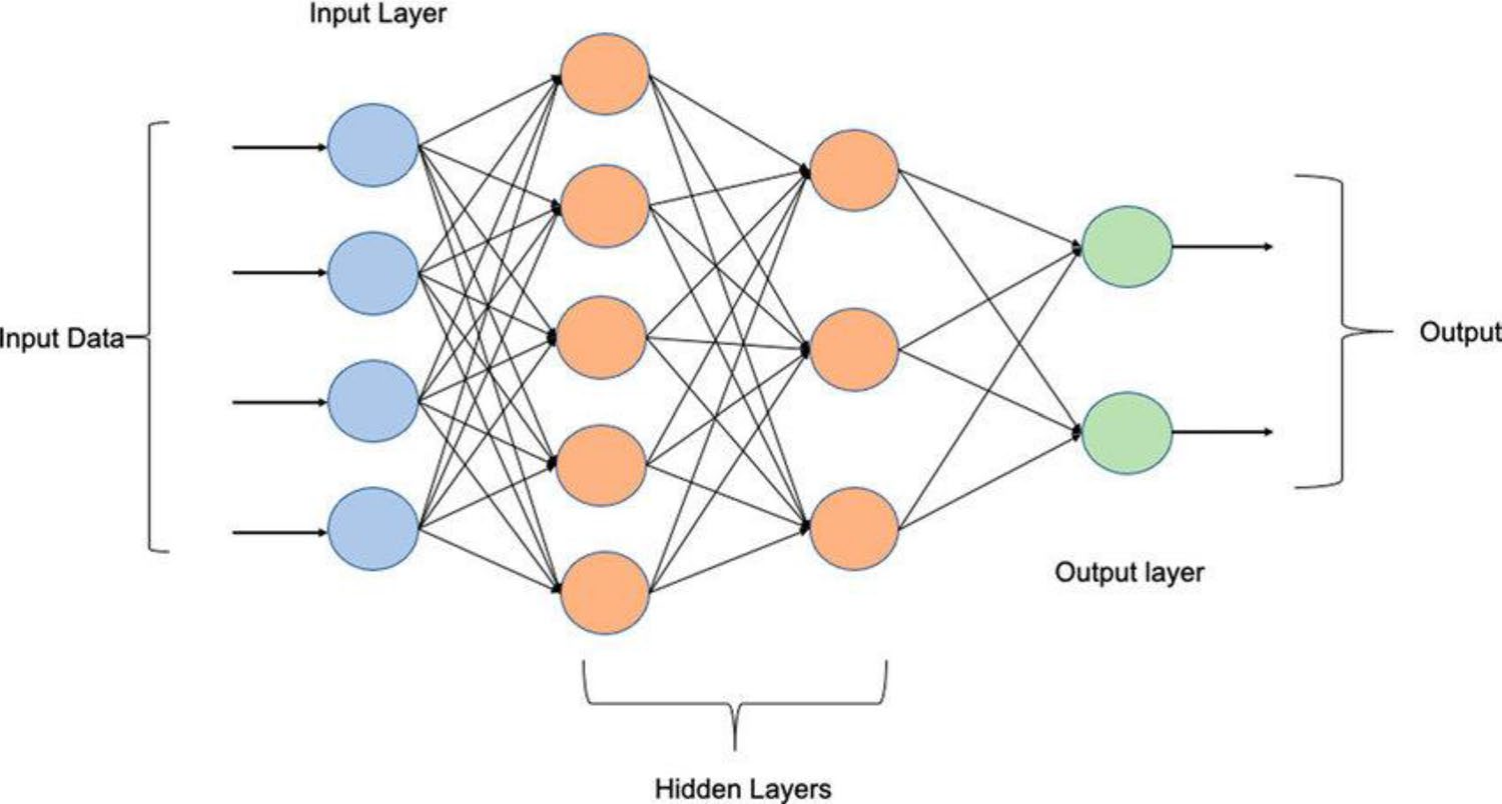
The ideal architecture of MLP network^54^.

A Multilayer Perceptron (MLP) is a fundamental type of artificial neural network (ANN) that consists of multiple layers of interconnected nodes, each performing computations on input data. The architecture typically includes an input layer, one or more hidden layers, and an output layer which is presented in Fig. 4^54^. MLPs are widely used for various machine learning tasks, such as classification, regression, and pattern recognition. Each node in the network is associated with a weight that modulates the influence of its input. The outputs of nodes are transformed using activation functions, which introduce non-linearity and enable the network to capture complex relationships in data. The formulation of an MLP involves the weighted sum of inputs at each node, followed by the application of an activation function^51^. This process propagates the signal through the network, transforming it as it passes through each layer. The weights of the connections are learned during the training phase by minimizing a chosen loss function, which measures the difference between predicted and actual outputs. Gradient descent algorithms, like the backpropagation algorithm, iteratively adjust the weights to minimize the loss function and improve the network's performance. The architecture of an MLP, including the number of layers, nodes in each layer, and activation functions, can be tailored to suit the specific task and dataset. More complex architectures, with additional hidden layers and nodes, allow the network to capture intricate patterns in data. Activation functions like ReLU introduce non-linearity, enhancing the network's ability to model complex relationships. However, designing an optimal architecture involves striking a balance between model complexity and overfitting. Hyperparameter tuning and experimentation play a crucial role in finding the right architecture for the given problem domain and dataset^52^.

Utilizing Multilayer Perceptron (MLP) for predicting Point Load Test Index (I_s_) in rocks provides distinct advantages within the realm of geotechnical engineering. MLP's adeptness at capturing intricate and non-linear relationships is particularly advantageous for understanding the complex interplay of factors influencing rock strength, as measured by Point Load Test Index. By automatically extracting relevant features from input data, MLPs enhance predictive accuracy, considering variables such as mineral composition, porosity, and sample diameter. This adaptability to diverse datasets ensures that the model can generalize well to various rock types, making it a versatile tool in geological contexts. Furthermore, MLP's data-driven approach leverages historical data to uncover nuanced dependencies that might elude traditional analytical methods. The improved accuracy resulting from MLP's pattern recognition capabilities contributes to more informed decision-making in geotechnical projects. Notably, the reduced need for resource-intensive physical testing through MLP predictions streamlines the testing process and enhances efficiency. Additionally, the model's ability to continuously learn and update with new data ensures its relevance in evolving geological conditions. In essence, the integration of MLP into Point Load Test Index prediction augments accuracy, efficiency, and adaptability in geotechnical assessments, offering valuable insights into the relationship between rock properties and strength.

Despite the advantages of utilizing Multilayer Perceptron (MLP) for predicting Point Load Test Index (I_s_) in rocks, there are certain limitations that need to be considered. One primary limitation is the reliance on a substantial amount of high-quality labeled data for training the model effectively. Insufficient or biased training data could lead to inaccurate predictions and compromise the model's reliability. Moreover, MLP's black-box nature can make it challenging to interpret the underlying reasons behind the model's predictions. This lack of interpretability might hinder the model's adoption in situations where clear explanations for the predictions are necessary for decision-making and analysis. Additionally, MLP's performance heavily depends on appropriate hyperparameter tuning, such as the number of hidden layers, nodes, and activation functions. Inadequate tuning might lead to overfitting or underfitting the model, affecting its generalization capability to unseen data. Another limitation arises from the assumption of stationarity in the data distribution; if the geological conditions or rock properties change significantly over time, the model's predictive performance might degrade. Furthermore, MLP's training and deployment require computational resources, which can be a concern in resource-limited environments. While MLPs offer valuable insights into the relationships between Point Load Test Index and rock properties, understanding these limitations is crucial for ensuring accurate and reliable predictions. Combining the strengths of MLPs with complementary approaches can help overcome these challenges and provide a more comprehensive understanding of rock behavior and strength.

The versatility of the multilayer perceptron (MLP) extends to both classification and regression tasks. In classification, the MLP is trained to categorize input data into predefined classes, while in regression, it is tasked with predicting continuous output variables based on one or more input variables^52^. A pivotal aspect of MLP lies in the selection of activation functions for its nodes. These functions introduce nonlinearity into the network, mapping a node's input to its corresponding output, thus empowering the network to capture intricate relationships between inputs and outputs. A range of activation functions holds sway within MLP's architecture. Notably, the sigmoid, hyperbolic tangent, Softmax, and rectified linear unit (ReLU) functions are recurrent choices, each contributing to the network's ability to process and interpret data^51^. These activation functions play a crucial role in endowing the MLP with its capacity to model complex patterns and nonlinear connections, rendering it a powerful tool in various data analysis tasks.

Rectified Linear Unit (ReLU) is an activation function commonly used in artificial neural networks, including the multilayer perceptron (MLP). It introduces nonlinearity by allowing the passage of positive values unchanged, while zeroing out negative inputs. Mathematically, ReLU(x) = max(0, x). This simplicity and computational efficiency have contributed to its popularity. ReLU addresses the vanishing gradient problem encountered with other activation functions like sigmoid and tanh, enabling faster convergence during training. However, ReLU can suffer from the "dying ReLU" problem, where some neurons get stuck and never activate. Variants like Leaky ReLU and Parametric ReLU have been introduced to mitigate this issue. The Softmax function, often used in the output layer of neural networks, is specifically employed for multiclass classification problems. It takes a vector of raw scores and converts them into a probability distribution, with each score representing the likelihood of an input belonging to a certain class. The Softmax function calculates the exponential of each score and then normalizes these values to sum up to 1. This ensures that the outputs can be interpreted as class probabilities. The class with the highest probability is the predicted class. Softmax enables the model to make decisions based on multiple classes and is commonly used in scenarios such as image classification and natural language processing tasks within the context of the MLP and other neural network architectures.

The integration of activation functions like ReLU and Softmax within the framework of neural networks, such as the MLP, is driven by their distinct advantages in addressing crucial challenges. ReLU, known for its computational efficiency and ability to introduce nonlinearity, proves essential in capturing complex relationships in data while mitigating the vanishing gradient problem. This accelerates learning in deep networks and promotes convergence. However, caution is exercised to counter the "dying ReLU" issue through variants like Leaky ReLU and Parametric ReLU. Meanwhile, Softmax assumes significance in multiclass classification, enabling the transformation of raw scores into interpretable probabilities. By facilitating informed decision-making among various classes and ensuring the output sums up to 1, Softmax greatly enhances the MLP's capability to make accurate predictions, especially in scenarios involving multiple classes like image classification and natural language processing.

### MLP model implementation

The construction of the MLP model was realized using the Keras framework. The input layer was fashioned to encapsulate the I_s_ information derived from a comprehensive main database encompassing 150 data entries. The model's outcome materialized in the output layer, visualizing either the rock's compressive strength or its UCS values. Within the MLP architecture, Dense layers were incorporated, wherein the initial layer comprised 64 hidden units, followed by a subsequent layer with 32 hidden units. To determine the behaviour of each layer, the activation parameter played a pivotal role, specifying the activation function applied at every stage. The ultimate layer was distinguished by the employment of the Softmax activation function, a fitting choice for multi-class classification challenges, all coded in the Python high-level programming language. For the optimization of model accuracy, the categorical cross-entropy loss function was adopted in conjunction with the Adam optimizer, a renowned technique adjusting the learning rate throughout the training process. This framework was employed to accommodate the specificities of multi-class classification tasks. Subsequently, the 'fit method' was employed, ushering the model through training iterations over a specified number of epochs (100 in this case) and employing a batch size of 32. To monitor the model's efficacy, the validation data parameter was introduced, facilitating the utilization of a distinct validation dataset during training.

The bedrock of this study comprised a principal database encompassing 150 distinct rock samples derived from Azarshahr travertine. This corpus was randomly partitioned, dedicating 70% for training and reserving the remaining 30% for testing. Consequently, the training dataset was enriched with 105 samples, while the testing dataset encapsulated the details of the remaining samples. The comprehensive depiction of the implemented MLP model's architecture and workflow is further illustrated as part of this endeavour. The process involves several crucial steps for implementing the MLP and evaluating its predictive prowess:

- Input data definition: commencing with the foundation, the input data for the MLP is established using a dataset encompassing 150 distinct rock samples from the primary database.
- Training and testing set separation: the division of data into training and testing sets is pivotal. The training set, containing 105 data entries, serves as the basis for training the model, while the testing set, comprised of the remaining 45 samples, functions as the means to assess the model's predictive performance.
- MLP architecture setup: the architecture configuration for the MLP on the training set is initiated. This encompasses determining the number of layers, nodes within these layers, and activation functions for optimal training. Additionally, criteria for evaluating the model's efficacy are defined, and a specific optimization algorithm, such as the Adam optimizer, is specified.
- Optimization algorithm definition: to guide the model's learning process, the loss function is meticulously defined. In this case, the cross-entropy loss function is selected. Simultaneously, the choice of the optimization algorithm, Adam optimizer, is made to facilitate the training process.
- Model validation on testing set: once the MLP completes its training, validation is conducted on the testing set, which comprises 45 remaining sample data points. The testing data is processed through the trained MLP, allowing a comparison between its predictions and the true labels, thereby gauging its predictive accuracy.
- Confusion matrix calculation: to assess the model's performance comprehensively, a confusion matrix is computed. This matrix serves as a comprehensive representation of the model's predictive capabilities, encompassing accuracy; precision, recall, and F1 score metrics, providing insights into its proficiency.
- Softmax activation for probabilities: lastly, the MLP's output is refined using the Softmax activation function. This function transforms the MLP's output into a set of probabilities, offering a nuanced interpretation of its predictions as a probability distribution across the various rock types.

By adhering to this systematic workflow, the MLP's potential as a predictive tool can be harnessed and evaluated effectively.

The training set is the larger portion of the data, comprising 70% of the total dataset. It is used exclusively for training your machine learning model. During the training process, the model learns to recognize patterns, relationships, and features within this dataset. A larger training set allows your model to better understand the underlying data distribution and adapt its parameters (weights and biases) accordingly. However, it is important to note that a large training set can also make the training process computationally more intensive. Also, the test set constitutes the remaining 30% of the dataset and is kept entirely separate from the training data. It is not used in any way during the model training process. Instead, the test set is reserved for evaluating the model's performance after training. Once your model is trained on the training set, you apply it to the test set to assess its ability to make accurate predictions on new, unseen data. The test set serves as an independent benchmark to measure how well your model generalizes to real-world scenarios, helping you gauge its effectiveness. The 70%-30% split is a common choice for dividing data into training and test sets. It strikes a balance between providing the model with enough data for learning and preserving a sufficiently large and independent set for evaluation. This separation ensures that the model's performance evaluation on the test set reflects its ability to handle new, unseen data, making it a crucial step in the machine learning workflow for estimating model performance and generalization.

The subsequent phase involves the systematic partitioning of the dataset into distinct subsets earmarked for training, validation, and testing. The training subset assumes a pivotal role in facilitating weight optimization during the iterative training process. The validation subset serves as a crucible for hyperparameter tuning and the prevention of overfitting. The testing subset, in turn, enables a robust evaluation of the trained DMLP model. Augmentation techniques, encompassing data augmentation, may be judiciously invoked to diversify the training dataset and enhance the model's generalization prowess. The applied hyperparameters in this study is provided in Table 2.

**Table 2:** A list of main hyperparameters that used in this study.

Algorithm	Hyperparameters	Values
MLP	Learning rate (learning_rate)	0.001
	Number of hidden layers (num_layers)	5 layer
	Number of neurons each Hidden Layer (hidden_units)	64
	Activation functions	
	ReLU	ReLU(x) = max(0, x)
	Sigmoid	Sigmoid(x) = 1/(1 + exp(− x))
	Batch size (batch_size)	32
	Dropout rate (dropout_rate)	0.2
	L2 regularization parameter (weight_decay)	λ = 0.001
	Epochs	1000
	Optimizer	Adam

Hyperparameters represent crucial configuration settings within machine learning algorithms, exerting a substantial influence over a model's performance and behaviour^52^. Unlike internal parameters acquired through training data, hyperparameters are predetermined by practitioners and guide the learning process. They control key aspects such as model complexity, regularization strength, and optimization strategies^51^. Skillful calibration of hyperparameters enables practitioners to strike a delicate equilibrium between a model's aptitude for discerning intricate data patterns and its ability to steer clear of overfitting or underfitting pitfalls. For instance, in deep neural networks, hyperparameters like layer count, neuron quantity within layers, and learning rate govern the network's depth, capacity, and convergence rate. Similarly, algorithms like decision trees involve hyperparameters such as maximum depth and minimum samples per leaf, influencing the tree's size and intricacy^52^. By judiciously tuning these hyperparameters, practitioners can tailor a model's behaviour to harmonize with specific problem nuances and dataset attributes, ultimately yielding optimal performance and enhanced generalization to previously unseen data^55^.

In this study, the MLP was employed as the primary machine learning technique for model training and predictive modelling. It is noteworthy that in traditional machine learning practices, the dataset is typically partitioned into three distinct subsets: the training set, validation set, and testing set. The training set is utilized for the actual model training, the validation set is essential for hyperparameter tuning and monitoring the model's performance during training and the testing set serves as an independent benchmark to assess the model's generalization capabilities to previously unseen data. However, one notable aspect in this study is the absence of explicit details regarding the specific allocation of data to the training and validation sets. To ensure transparency, reproducibility, and clarity in the methodology employed, it is imperative that the author specifies the size or proportion of the dataset assigned to the validation set. This precision is critical for comprehending the model development process, facilitating readers' understanding of dataset utilization, and allowing for the replication of experiments. Therefore, while the paper effectively introduces the utilization of MLP in predictive modelling, enhancing the manuscript with explicit information regarding the dataset split between the training and validation sets would substantially augment the study's methodological rigor and transparency, aligning it with established academic practices.

### Model validation and verification

**Figure 5:**
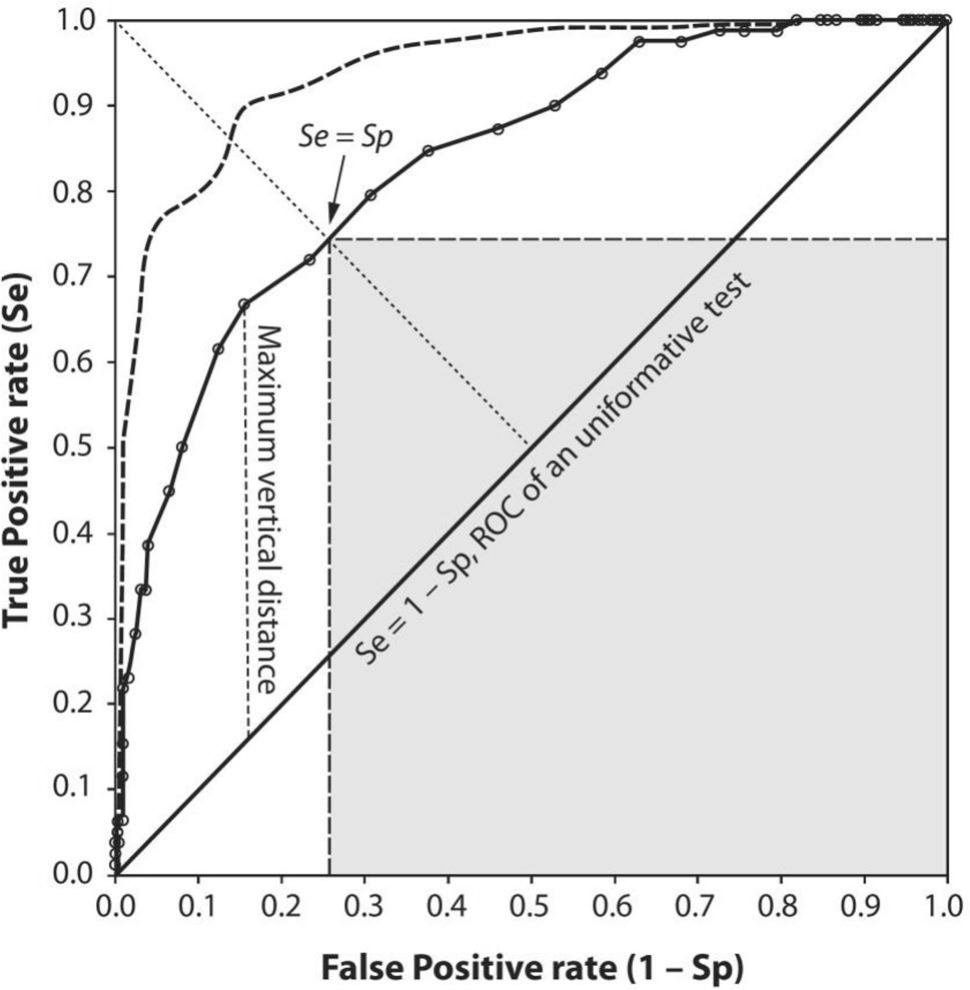
The topical ROC curve for analysis (adapted from^56^).

To ascertain the predictive model's susceptibility to overfitting or underfitting, it becomes imperative to evaluate its performance on data that hasn't been encountered during its training phase. In this context, the presented study leveraged two distinct validation approaches: the train/test split method and cross-validation techniques. In the train/test split approach, the primary database was randomly divided into training and testing subsets, enabling the assessment of the confusion matrix for both sets. On the other hand, the cross-validation procedure involved the application of receiver operating characteristic (ROC) curve analysis on the datasets, fostering a comprehensive comparison of outcomes^51^. The ROC curve graphically illustrates the performance of a binary classification model across varying classification thresholds, showcasing the trade-off between True Positive Rate (TPR) and False Positive Rate (FPR). The topical ROC curve was illustrated in Fig. 5. In this figure, the ROC curve illustrates a comparison between two curves, one dashed and one solid. The dashed curve entirely lies above the solid curve, indicating a superior test with a larger area under the curve. The left-upper corner of the ROC curve, represented by the solid line, moves along it. The maximum area of the shaded rectangular region occurs when the sides of the rectangle, denoted as sensitivity (Se) and specificity (Sp), are equal. Sensitivity refers to the ability to correctly identify positive cases, while specificity pertains to the capacity to correctly identify negative cases^56^.

In the realm of ROC analysis, the binary classification entails two classes, commonly termed positive (1) and negative (0). For each class, the classifier generates probability predictions, with a decision threshold dictating sample classification as positive or negative. Plotting TPR against FPR at different threshold values yields the ROC curve, providing insights into the model's behaviour. TPR represents the proportion of correctly identified positive samples, while FPR signifies the proportion of incorrectly identified negative samples. Widely employed in medical diagnosis, fraud detection, and credit scoring, the ROC curve is pivotal for assessing binary classifier performance across diverse thresholds. The curve associated with a perfect classifier aligns with the top-left corner, whereas a random classifier's curve follows the diagonal line. The area under the ROC curve (AUC) serves as a common metric to gauge a binary classifier's overall performance, ranging from 0.5 (random classifier) to 1 (perfect classifier)^52^. Employing these rigorous validation methods effectively equips the implementation of predictive models, mitigating the risks of overfitting or underfitting phenomena.

To ascertain the consistency between prediction and measurement outcomes within the primary database, the analysis employed the coefficient of determination, commonly referred to as R-squared (R^2^). This statistical parameter serves as a pivotal tool for cross-validation, quantifying the proportion of variability in the dependent variable elucidated by the independent variables in a given regression model. In essence, R^2^ gauges the efficacy of the regression model in aligning with the observed data, effectively measuring the degree of fit between the model and the dataset under scrutiny^51^.

### Model justifications

In evaluating the performance of the presented predictive model, a comparative analysis was conducted. In this context, traditional machine learning approaches, including random forests (RF), decision trees (DT), logistic regression (LR), support-vector machines (SVM), and k-nearest neighbours (k-NN), were considered for comparison. RF is an ensemble learning method that combines multiple decision trees to make predictions. It works by creating a multitude of decision trees during training and then averaging their predictions during testing. This ensemble approach tends to reduce overfitting and improve accuracy, making it particularly useful for classification and regression tasks. DT is a simple yet powerful machine learning algorithm used for both classification and regression tasks. They partition the data into subsets based on feature values, creating a tree-like structure. Each internal node represents a decision based on a feature, and each leaf node represents the predicted class or value. Decision Trees are interpretable and can handle both categorical and numerical data. LR is a widely used classification algorithm. It models the probability that a given input belongs to a particular class using the logistic function. Despite its name, logistic regression is used for classification, not regression. It's simple, interpretable, and works well for binary and multiclass classification problems. SVM is a powerful classification algorithm that aims to find a hyperplane that best separates data into different classes. It works by finding the maximum-margin hyperplane, which maximizes the margin between the closest data points of different classes. SVMs can handle linear and non-linear classification problems and are effective for both binary and multiclass tasks. k-NN is a straightforward classification algorithm that classifies a data point based on the majority class among its k-nearest neighbors in the feature space. It's a non-parametric and instance-based algorithm, meaning it doesn't make strong assumptions about the underlying data distribution. However, it can be sensitive to the choice of k and may not perform well with high-dimensional data^51,52^.

In the context of this study, the inclusion of traditional machine learning classifiers, including RF, DT, LR, SVM, and k-NN, stands as a critical component of a rigorous comparative analysis. This methodological approach allows for a comprehensive evaluation of diverse machine learning algorithms, facilitating an informed selection process for the most apt modelling technique tailored to the specific predictive task at hand. It serves as a benchmarking exercise, permitting a thorough assessment of the efficacy and appropriateness of the MLP-based predictive model relative to well-established, conventional methodologies. The comparative analysis undertaken herein is instrumental in elucidating several pivotal aspects. Firstly, it offers insights into the intricacies of model complexity, as various algorithms encompass a spectrum ranging from simplicity, exemplified by DT and LR, to heightened complexity embodied by RF and MLPs. This assessment aids in addressing paramount questions concerning the necessity of employing a complex model like MLP in relation to simpler alternatives. Moreover, the comparative approach affords a comprehensive exploration of model robustness and generalization capabilities, shedding light on the resilience of each classifier to data anomalies and their ability to extrapolate effectively to unseen data. Furthermore, it provides an opportunity to strike a balance between model interpretability, vital in certain applications, and predictive performance. Finally, this approach facilitates the discernment of how each classifier copes with distinct data characteristics, aiding in the selection of the most suitable model based on factors such as feature space dimensionality and non-linear relationships within the dataset. In summation, the inclusion of traditional classifiers in this study is integral to making informed decisions pertaining to the optimal modelling approach, underpinning the study's scientific rigor and depth of analysis.

## Results and discussion

Upon completion of the point-load tests in accordance with the guidelines laid out by the ISRM regulations^11^ as stipulated in^31^, all relevant features were meticulously measured and documented. The tabulated data, delineated in Tables 3, forms the basis for ensuing statistical analysis. To facilitate the subsequent utilization of this data in MLP modeling, a crucial normalization step was executed. This entailed scaling the data within the range of 0 to 1, thereby ensuring equitable consideration for every variable during the training phase of the model. For this study, a linear transformation approach was adopted for feature scaling, effectuating the alignment of feature values within a consistent range. This meticulous process of feature scaling holds particular significance within machine learning models, especially those reliant on distance computations or gradient descent algorithms. As encapsulated by Eq. (4), this preprocessing step plays an indispensable role in enabling models to perform optimally by maintaining commensurate scales for input and output features.

4 $$ \overset{\lower0.5em\hbox{$\smash{\scriptscriptstyle\frown}$}}{y} = \frac{{y_{i} - y_{\min } }}{{y_{\max } - y_{\min } }} $$

where ŷ is standardised value for each feature at y set, y_i_ is extracted recorded value at point i, y_max_ and y_min_ are maximum and Minimum values in y set.

**Table 3:** A summary of statistics analysis for datasets.

Parameter	Max	Min	Mean	St.Dv.	Kurtosis	Skewness
Density (kN/m^3^)	25.47	25.16	25.30	0.0823	− 1.426	0.0137
I_s_ (axial)	2.36	1.13	1.829	0.3520	− 1.010	− 0.2860
I_s_ (diametral)	2.43	1.14	1.79	0.3461	− 1.001	− 0.2509
UCS_axial_	65.2	34.5	51.73	8.7504	− 0.985	− 0.3113
UCS_diametral_	65.0	34.5	50.28	8.5544	− 1.011	− 0.2584

*St.Dv.* standard deviation.

As depicted in Table 3, an index statistical analysis has been conducted on the prepared database, encompassing various input and target variables for this specific task. The database's evaluated variables have undergone a thorough statistical analysis, encompassing key metrics such as maximum, minimum, mean, standard deviation, kurtosis, and skewness. These statistical functions provide valuable insights into the distribution and characteristics of the dataset. The statistical analysis conducted on the evaluated variables within the database encompassed several key metrics. These metrics included the maximum, minimum, mean (average), standard deviation, kurtosis, and skewness. The maximum represented the highest observed value, while the minimum denoted the lowest. The mean, or average, portrayed the central tendency of the dataset by calculating the sum of all values divided by the total count. Standard deviation provided insight into the spread or variability of data points around the mean. Additionally, kurtosis indicated whether the data distribution had heavier or lighter tails compared to a normal distribution, and skewness revealed the dataset's asymmetry. These statistical measures played a crucial role in characterizing the dataset's properties and understanding its distribution patterns. Based on the information presented in the table, we can observe the range of variation among different input data attributes such as density (kN/m^3^), I_s_, and UCS (MPa) which exhibit variability within the dataset. The recorded values span from a minimum of 25.26 for density, 1.13 for I_s_, and 34.5 for UCS, to a maximum of 25.47 for density, 2.43 for I_s_, and 65.5 for UCS. On average, the dataset demonstrates typical values of approximately 25.30 for density, 1.82 for I_s_, and 51.73 for UCS. These statistics provide a clear overview of the dataset's range, central tendency, and dispersion, which are crucial for understanding the underlying patterns and characteristics of the data.

Statistical analysis is pivotal for our work due to several key reasons. Firstly, it enables a comprehensive understanding of data distribution, shedding light on the behavior and patterns within various input attributes like density, I_s_ (both axial and diametral), and UCS (both axial and diametral). Secondly, it serves as a robust tool for data validation, crucial for identifying outliers or inconsistencies that might affect result reliability. Moreover, statistical analysis aids in the selection of relevant features, guiding us to focus on the attributes with the most significant impact on our target variable, UCS. Additionally, it provides a means to assess the performance of predictive models by employing metrics like R-squared (R^2^) to gauge model fit and effectiveness. Lastly, this analysis can unearth valuable insights or correlations within the data, offering potential directions for further research and a deeper understanding of variable relationships. In summary, statistical analysis is foundational in our data-driven research, ensuring data quality, aiding in modeling, and facilitating informed decision-making. R-squared (R^2^) holds significant importance in our research, where we aim to predict the unconfined compressive strength (UCS) of rock samples using the I_s_. R^2^ serves as a critical metric for assessing the quality and effectiveness of our predictive models. Essentially, it quantifies how well our models fit the observed data. A high R^2^ value indicates that our models successfully capture the variability in UCS based on the I_s_ values, suggesting a strong and accurate relationship. Moreover, R^2^ assists us in model selection and configuration. By comparing R^2^ values among different model variations, architectures, or hyperparameters, we can pinpoint the model setup that best suits our dataset. This ensures that our predictive models are optimized to provide the most accurate UCS estimates. Furthermore, R^2^ plays a pivotal role in evaluating the models' ability to generalize beyond the training data. Achieving a high R^2^ on the training dataset is just the first step. To ensure practical applicability, we must also attain a high R^2^ on the testing dataset, demonstrating that our models can make reliable UCS predictions for new, unseen rock samples.

**Figure 6:**
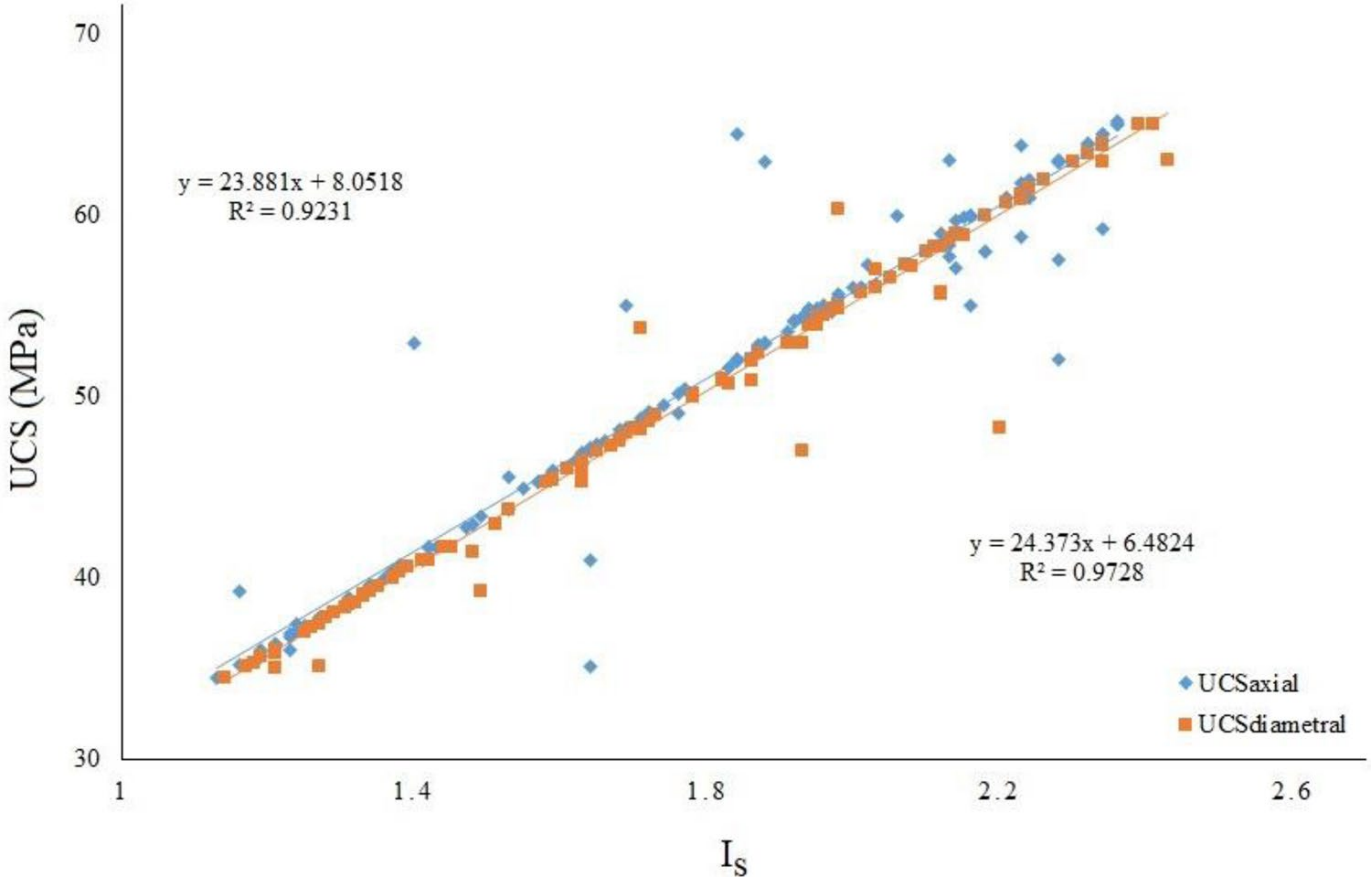
Empirical relationship between UCS and I_s_ for studied rocks.

Prior to the integration of the MLP model for forecasting UCS values based on the I_s_ parameter, a fundamental step entailed estimating the empirical relation tailored to Azarshahr travertine. This endeavor involved the application of linear regression analysis, coupled with the assessment of the R^2^ coefficient. This coefficient serves as a quantifiable indicator of the model's explanatory power. The empirical relation was strategically deduced for both axial and diametral point-load strength indexes. The outcome of this analytical process is vividly depicted in Fig. 6, where the empirical relation curated for the meticulously compiled database is visually showcased. The insights derived from this graphical representation yield an estimated R^2^ coefficient of 0.9231 for axial strength and an even more impressive 0.9728 for diametral strength in relation to UCS values. This substantiates the alignment of the empirical relation with the observed data and underscores its potential utility in predictive UCS modeling.

**Figure 7:**
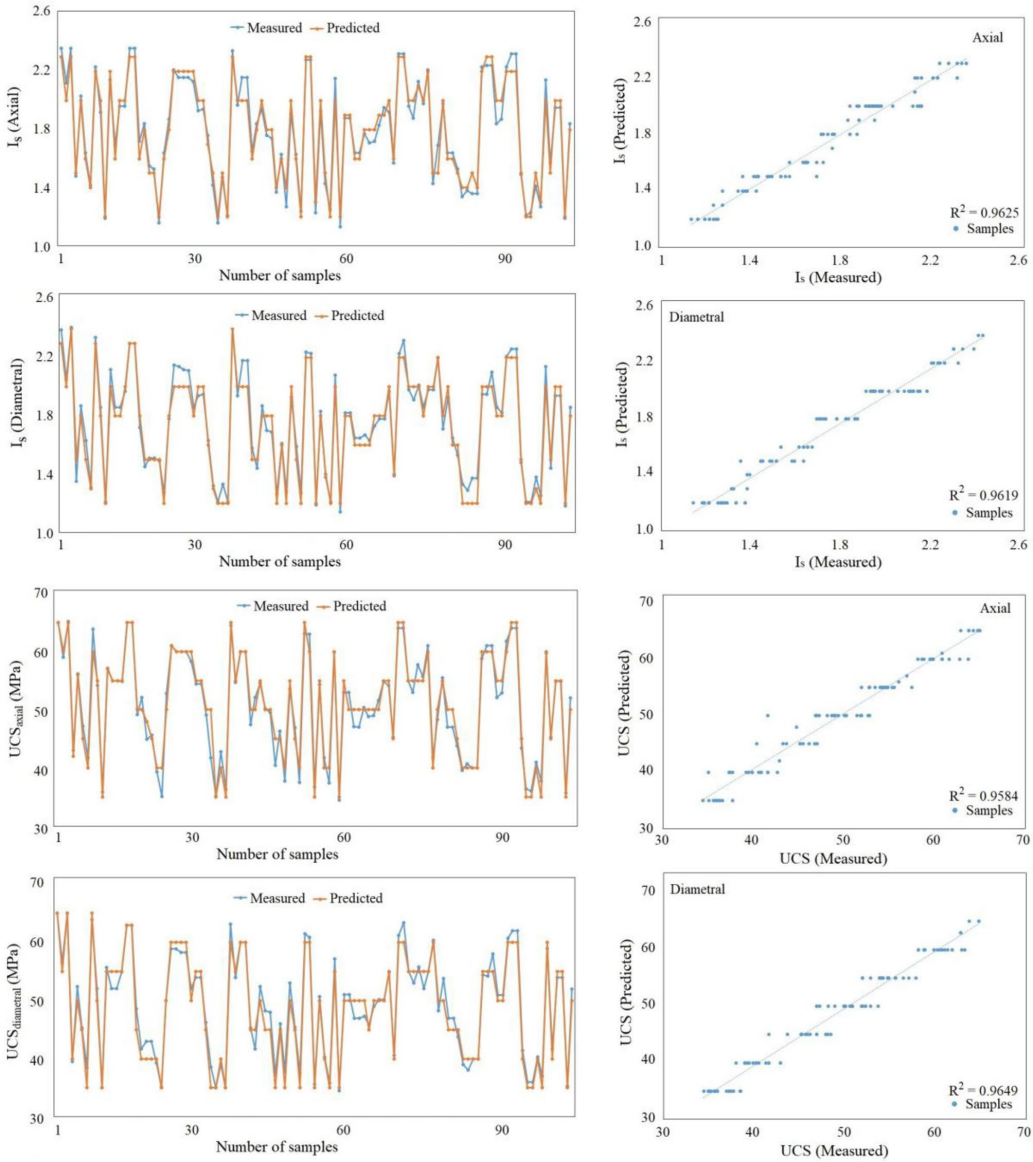
Comparison of measured and predicted UCS for travertine rocks in training dataset.

**Figure 8:**
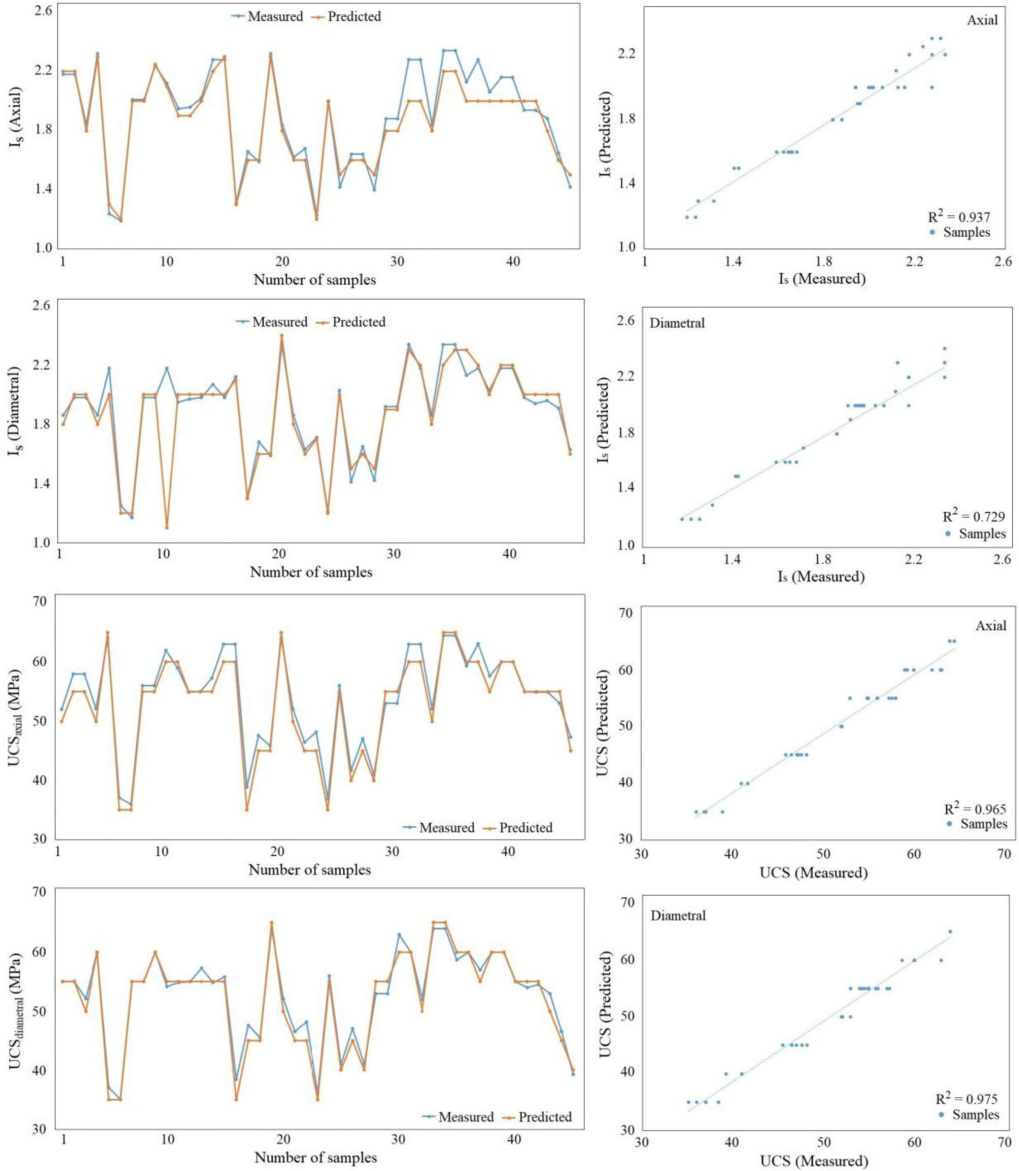
Comparison of measured and predicted UCS for travertine rocks in testing dataset.

Following the establishment of empirical correlations between UCS and I_s_ for the investigated rock samples, this data was harnessed to forge predictive models through the MLP classifier. Operating on the principles of supervised learning, the entire dataset underwent meticulous labeling, with features systematically categorized as per ISRM guidelines. The MLP model was subsequently executed, and the resultant predictions for UCS were recorded. This performance was meticulously assessed, with the outcomes showcased through Figs. 7 and 8. These illustrative visuals serve to highlight the contrasting dynamics between the measured and forecasted values attributed to the MLP model. The comprehensive comparison encapsulated in these figures extends across both the testing and training datasets, thereby affording a holistic insight into the predictive prowess of the MLP model in diverse scenarios.

After deriving empirical relationships connecting Uniaxial Compressive Strength (UCS) and the Point Load Index (Is) for the examined rock specimens, this dataset was utilized to construct predictive models using the Multilayer Perceptron (MLP) classifier. Employing the principles of supervised learning, the complete dataset underwent meticulous labeling, with features methodically categorized according to guidelines outlined by the International Society for Rock Mechanics (ISRM). Subsequently, the MLP model was implemented, producing forecasts for UCS values. The performance of this model underwent thorough evaluation, with the results visualized through Figs. 7 and 8. These graphical representations effectively emphasize the contrasts between the actual and predicted values generated by the MLP model. By encompassing both the testing and training datasets, these illustrations provide a comprehensive understanding of the MLP model's predictive capabilities across various scenarios. In the graphical depictions presented in Figs. 7 and 8, the observed discrepancies between the actual UCS values and the UCS values predicted by the MLP model are evident. This visual comparison not only underscores the model's performance but also enables a quick assessment of its accuracy. The graphs unveil trends, potential outliers, and the overall alignment between predictions and real values. This comprehensive representation is particularly beneficial in decision-making processes, offering insights into the model's reliability and potential areas of improvement. Furthermore, the inclusion of both testing and training datasets in the graphical analysis enables a thorough assessment of the model's generalization ability. Overfitting or underfitting issues become more apparent when the model's performance on unseen data is compared to its performance on the training data. These figures allow researchers and practitioners to gauge whether the MLP model successfully captures the underlying patterns in the data without overemphasizing noise or specific cases encountered during training. Overall, these graphical representations encapsulate the entire evaluation process, showcasing the strengths and limitations of the MLP model in predicting UCS values based on Is. These visuals provide valuable insights into the model's predictive prowess and its ability to generalize to new data, guiding future refinements and enhancements to improve its performance.

In the realm of machine learning and model validation, cross-validation is a well-established practice used to evaluate a model's performance. Conventionally, this approach involves partitioning a training dataset into subsets, training the model on different combinations of these subsets, and assessing its performance across various iterations. However, the present study deviates from this customary procedure by employing ROC analysis as a form of cross-validation (70%-30% in this study). The rationale behind this unconventional choice lies in the specific problem addressed within the study and its associated research objectives. The core aim of the research is to predict I_s_ and UCS values for rock samples utilizing the point-load index. Given that ROC analysis is particularly adept at evaluating the performance of binary classifiers, it aligns seamlessly with the essence of the research problem. In essence, the MLP model employed in this study undertakes binary classification by determining whether a given rock sample belongs to a specific category based on its Is value. This underlying binary classification nature necessitates the utilization of ROC analysis, which enables a comprehensive assessment of the model's sensitivity and specificity across various classification thresholds. Consequently, ROC analysis empowers the study to gauge the MLP model's capacity to effectively discriminate between different sample classes and make precise predictions, ultimately harmonizing with the overarching research objective of UCS estimation from I_s_ values. In summary, while conventional cross-validation methodologies are pervasive, the selection of ROC analysis as a cross-validation technique in this study is judiciously motivated by the unique binary classification character of the research problem. This analytical approach not only validates the model's performance but also accentuates its suitability for discerning between various sample classes, consequently enriching the study's ability to predict UCS values based on the I_s_ index.

**Figure 9:**
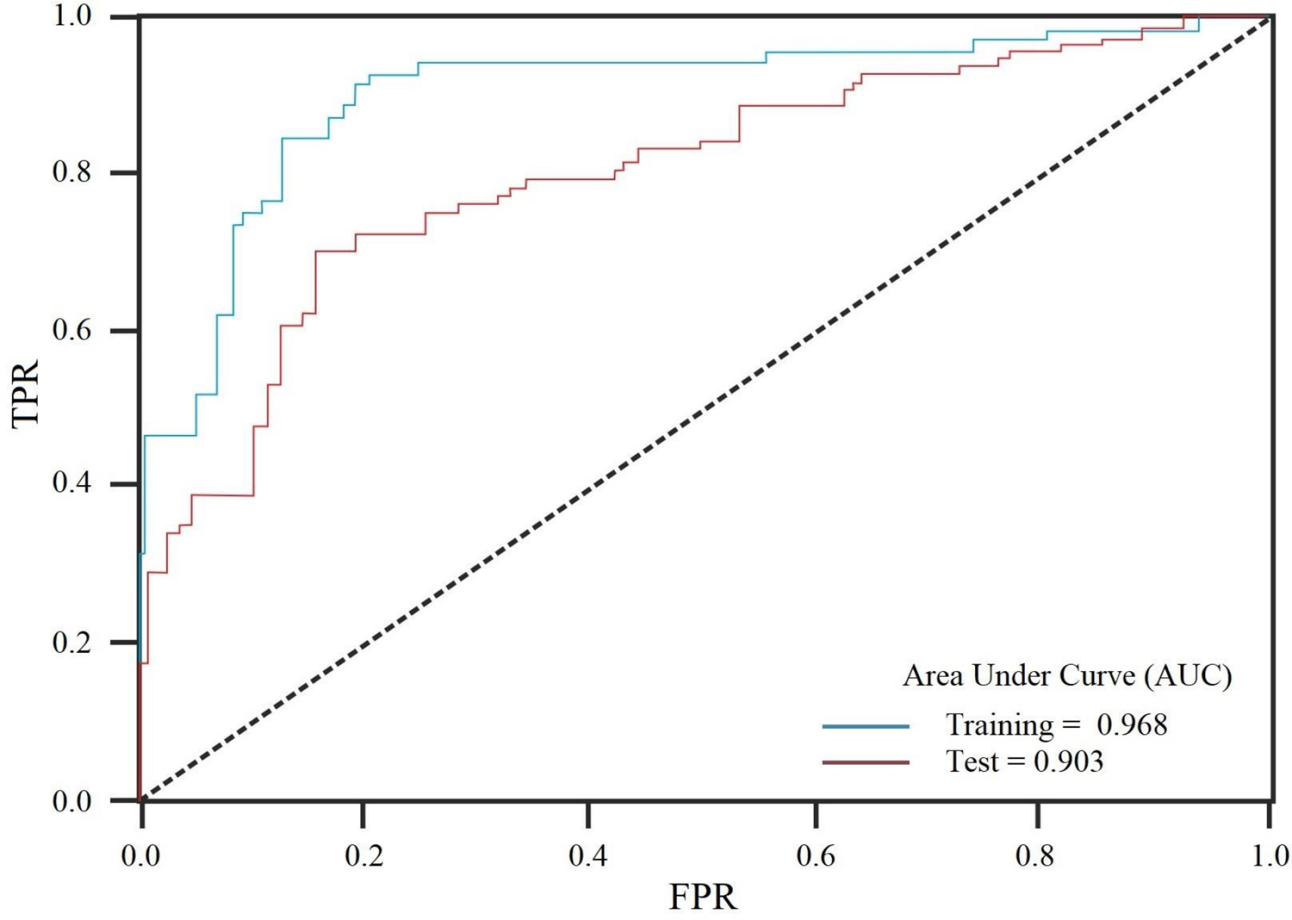
ROC curve analysis for model’s capability evaluation.

Subsequent to an exhaustive training regimen employing the training dataset, the efficacy of the model underwent rigorous assessment through the scrutiny of a confusion matrix, which is encapsulated within Table 4. The discernible results emerging from this comprehensive performance analysis underscore the attainment of model accuracy reaching an impressive 0.96 and 0.90 for the training and testing datasets, respectively, indicative of noteworthy predictive capabilities. To augment this evaluation, Fig. 9 offers a visual exposition of the ROC analysis findings derived from both the training and testing datasets, thereby facilitating the estimation of the overall accuracy (OA). Delving deeper, the ROC curve delineates that the OA for the training dataset attains a value of 0.968 (AUC), while the OA for the test dataset stands at 0.903 (AUC). This substantiates the ROC's pivotal role in corroborating the outcomes of the confusion matrix, attesting to the model's adeptness in proficiently predicting Is and UCS values with commendable accuracy and a high degree of reliability.

**Table 4:** Results of performance analysis based on confusion matrix for applied model.

Dataset	Evaluation criteria			Accuracy
	Precision	Recall	F1-score	
Training	0.97	0.96	0.96	0.96
Testing	0.90	0.87	0.90	0.90

The ROC curve visually depicts the relationship between the true positive rate (sensitivity) and the false positive rate (1-specificity) as the classification threshold changes. A higher AUC value signifies better model performance, with values closer to 1 indicating excellent predictive accuracy. Therefore, the AUC values obtained in this study indicate that the model excels in distinguishing between different rock conditions based on point-load index and accurately predicting their UCS values. These results bolster the confidence in the model's ability to reliably forecast rock strength properties using the point-load index. The combination of the confusion matrix and ROC curve analysis provides a comprehensive assessment of the model's predictive capabilities, validating its potential for real-world applications where accurate estimations of rock strength are essential for decision-making processes in various fields such as mining and geotechnical engineering.

As previously outlined, a diverse array of traditional machine learning techniques, spanning RF, DT, LR, SVM, and k-NN, was employed to facilitate a comprehensive comparative assessment of the MLP-based model's performance. Notably, these classifiers were applied to the same dataset using an identical split ratio, with 70% allocated for the training set and 30% for the testing set. This meticulous and standardized comparative analysis was paramount in discerning the efficacy and applicability of the MLP-based model within the context of well-established conventional methodologies. Table 5 presents the outcomes derived from the comparative predictive models, as assessed through confusion matrix. As evident from the table, the MLP model achieved the highest levels of accuracy and precision compared to the other classifiers in the study. This underscores the superior performance of the MLP-based approach.

**Table 5:** Results of performance analysis for justification classifiers.

Classifier	Evaluation criteria			Accuracy
	Precision	Recall	F1-score	
MLP (training)	0.97	0.96	0.96	0.96
FR	0.63	0.65	0.65	0.63
DT	0.68	0.65	0.65	0.68
LR	0.64	0.67	0.64	0.64
SVM	0.80	0.83	0.83	0.80
k-NN	0.75	0.77	0.75	0.77

The process of model training is a critical phase in machine learning, and in this context, the model's proficiency was rigorously evaluated through a confusion matrix analysis, the results of which are succinctly presented in Table 3. This analytical tool systematically portrays the model's classification performance, showcasing the instances of true positives, true negatives, false positives, and false negatives. Such an in-depth evaluation is imperative as it provides a comprehensive snapshot of how well the model can differentiate between different classes, thereby offering insights into its prediction accuracy and potential areas of improvement. By scrutinizing the confusion matrix, we gain a nuanced understanding of the model's strengths and weaknesses, paving the way for informed decisions on refining the model's architecture and parameters for optimal performance.

Turning our attention to the performance analysis, the derived outcomes yield noteworthy conclusions. The model's prowess is exemplified by the achieved accuracy rates of 0.96 for the training dataset and 0.90 for the testing dataset. These percentages depict the proportion of correctly predicted instances, indicating a high level of predictive competency. This level of accuracy is particularly impressive considering the inherent complexities within the dataset and the inherent challenges in modeling rock strength predictions. Such results resonate with the practical implications of the model, as high accuracy is imperative in domains where accurate predictions hold significant value, such as geological and engineering applications.

The assessment was further enriched by the incorporation of ROC (Receiver Operating Characteristic) curve analysis, an established technique for evaluating classification model performance across varying thresholds. Figure 9 encapsulates the ROC curve outcomes, offering an insightful visualization of the trade-off between true positive rate (TPR) and false positive rate (FPR). This graphical representation enhances our understanding of how the model performs across different classification scenarios. With an AUC (Area Under the Curve) value of 0.968 for the training dataset and 0.903 for the testing dataset, the ROC analysis underscores the model's consistency in distinguishing between classes, emphasizing its robustness and efficacy in making accurate predictions.

In a broader context, the confluence of the confusion matrix analysis and the ROC curve assessment bolsters our confidence in the predictive model's reliability and applicability. The agreement between these analyses corroborates the model's ability to accurately forecast both Is and UCS values. As the ROC analysis aligns with the confusion matrix results, it substantiates the notion that the model's predictions are both accurate and reliable. Moreover, the ROC analysis reaffirms the model's suitability for real-world applications where reliable predictions are paramount. Overall, the meticulous evaluation not only sheds light on the model's current performance but also lays the foundation for future refinements and enhancements to amplify its predictive capabilities across varied contexts.

The application of MLP models in point-load prediction for rocks present notable advantages and considerations, as reflected in the ROC and AUC outcomes. Demonstrated by AUC values of 0.968 for training and 0.903 for testing, the MLP model excels in accuracy, leveraging its capacity to grasp intricate data relationships. Its predictive prowess empowers precise categorization of diverse rock conditions, with potential implications for geotechnical engineering and mining sectors. However, the promising performance also entails certain limitations. The risk of overfitting looms, potentially compromising the model's generalization beyond the training set. A vigilant approach to data quality is paramount, as the model's effectiveness hinges on the comprehensiveness and representativeness of the dataset. Moreover, the complexity of MLP models poses challenges in understanding their internal mechanics, impeding interpretability—particularly significant in fields valuing transparent decision-making. Thus, while MLP offers potent predictive capabilities, a balanced assessment of its benefits and limitations is crucial for deploying it effectively in real-world applications.

Leveraging deep learning techniques, specifically deep Multilayer Perceptron (MLP), in point-load testing for rocks offers multifaceted advantages. Deep MLP has the capacity to uncover intricate patterns and relationships within complex datasets, enabling it to capture nuanced variations in rock properties that may elude conventional methods. Its hierarchical architecture allows it to automatically learn relevant features, reducing the need for manual feature engineering. This empowers the model to adapt and perform well even in scenarios with diverse rock types, unearthing predictive insights that can enhance drillability estimations and geotechnical decision-making. Additionally, the scalability of deep MLP enables it to handle vast and diverse datasets, accommodating a broad spectrum of rock samples. The model's ability to generalize across different geological contexts enhances its applicability beyond specific regions, supporting standardized point-load predictions globally. Furthermore, the adaptability of deep MLP empowers it to refine predictions over time as new data becomes available, fostering continuous improvement in accuracy. By harnessing the power of deep learning, particularly deep MLP, in point-load analysis, the geotechnical and mining industries stand to benefit from more precise, efficient, and adaptable predictive models that advance their understanding and management of rock properties.

## Conclusion

The current study endeavors to introduce a novel MLP-based predictive model geared towards prognosticating the compressive strength (UCS) of Azarshahr travertine, contingent on the point-load testing index (Is). The principal aim of this modeling endeavor is to devise a non-destructive avenue for estimating UCS predicated on limited I_s_ values, a salient asset in formulating drillability plans for open-pit mining ventures. To underpin this undertaking, an extensive database of 150 meticulously documented point load test records was meticulously assembled, meticulously adhering to the guidelines outlined by ISRM for both axial and diametral testing methodologies. Notably, the empirical strength regression analysis rendered an estimated R2 coefficient of 0.9231 for axial strength and an even more commendable 0.9728 for diametral strength in relation to UCS. This predictive model's efficacy was subjected to rigorous validation procedures, encompassing both train/test split and cross-validation methodologies. The former entailed randomly partitioning the provided database into training (70%) and testing (30%) subsets, enabling the MLP model to be meticulously honed and meticulously scrutinized. The model's accuracy was meticulously gauged through the lens of the confusion matrix, yielding impressive accuracy rates of 0.96 and 0.90 for the training and testing datasets, respectively. The robustness of the model's predictive potential was further accentuated through ROC curve analysis. This statistical tool, shedding light on the interplay between the true positive rate (TPR) and the false positive rate (FPR), unveiled an overall accuracy (OA) represented by AUC values of 0.968 for the training phase and 0.903 for the testing phase. In conclusion, the unveiled MLP-based predictive model stands as a pioneering contribution, underpinned by empirical relations, meticulous training, and comprehensive validation. Its adeptness in forecasting UCS values from limited Is indices ushers in a realm of possibilities for enhanced drillability planning in open-pit mining endeavors. By bridging the gap between empirical relations and data-driven predictions, this model bears significant promise in refining excavation strategies and bolstering mining operations.

As a stepping stone for future endeavors, the current methodology offers a solid foundation for further exploration and enhancements. To extend the applicability and robustness of the MLP-based predictive model, integrating a broader range of geological and environmental variables could yield insights into more intricate rock behavior patterns. Additionally, expanding the dataset to encompass diverse rock types and mining regions would fortify the model's generalization capabilities. Exploring the potential of hybrid models, amalgamating different machine learning techniques, could unveil synergistic effects that enhance accuracy and predictive power. Moreover, the integration of real-time monitoring data from mining operations could facilitate continuous model calibration and refinement, ultimately resulting in more precise and responsive predictions. Lastly, collaboration with domain experts and stakeholders would ensure that the model aligns seamlessly with practical mining applications, fostering a comprehensive framework for optimized drillability planning and excavation strategies.

## Data Availability

All datasets or analysed process during this study is included in this published article.
